# Atopic Dermatitis Activity Score 7 (ADAS7): A tool for disease activity assessment

**DOI:** 10.1002/clt2.12393

**Published:** 2024-09-12

**Authors:** Axel De Greef, Alexia Degraeuwe, Nina Nielens, Anne‐Sophie Darrigade, Céline Bugli, Laurence de Montjoye, Marie Baeck

**Affiliations:** ^1^ Department of Dermatology Cliniques universitaires Saint‐Luc Université catholique de Louvain (UCLouvain) Brussels Belgium; ^2^ Institute of Experimental and Clinical Research (IREC) Pneumology, ENT and Dermatology Pole (LUNS) Université catholique de Louvain (UCLouvain) Brussels Belgium; ^3^ Plateforme Technologique de Support en Méthodologie et Support Statistique (SMCS) Université catholique de Louvain (UCLouvain) Louvain‐la‐Neuve Belgium

To the Editor,

The Harmonizing Outcome Measures for Eczema (HOME) group has established that the evaluation of patients with atopic dermatitis (AD) should measure clinical signs, patient‐reported symptoms, long‐term control of the disease, and patients' quality of life.^1^ To date, the existing scores^2^ often assess only one of the aspects and are time‐consuming. Furthermore, these scores assess the severity only at a certain time‐point or over a maximum of seven consecutive days, and do not take into account the disease *activity*, defined as the fluctuations of AD.^3^ We stated the need for a score that effectively assesses this dynamic aspect of the disease.^4^ Preliminary results of a newly developed score, the “Atopic Dermatitis Score 7” (ADS7), inspired by the Urticaria Activity Score 7 (UAS7), showed a good correlation with the scores currently used in AD (Figure 1).^5^

**FIGURE 1:**
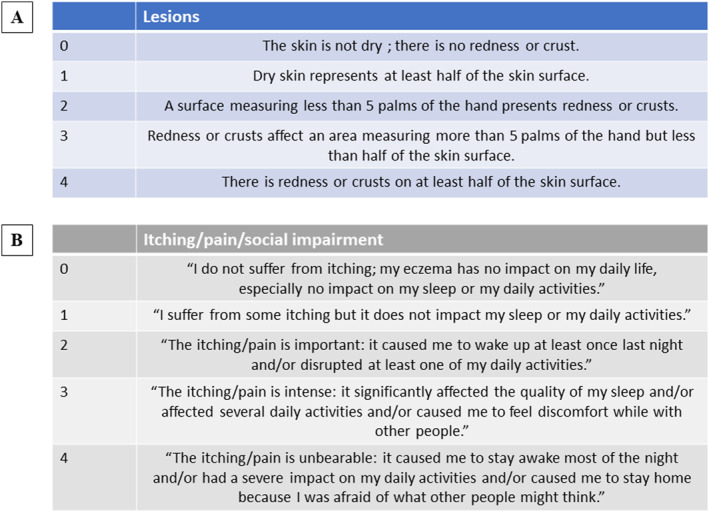
Atopic Dermatitis Activity Score 7 (ADAS7). The patient fills in the daily chart with a score from 0 to 4 for the body surface involved and severity of the eczema (A), and for itch, pain, and social impairment (B).

The aims of the present study were (i) to demonstrate ADS7 correlation with SCORing Atopic Dermatitis (SCORAD) on a larger cohort, and (ii) to evaluate if ADS7 was able to highlight the *activity* of the disease. It was therefore renamed “Atopic Dermatitis Activity Score 7” (ADAS7). We prospectively enrolled 137 patients with AD between September 2021 and August 2023 from Belgian academic and non‐academic hospitals (outpatient clinics). One hundred patients completed the score daily for a period of maximum 6 months and were included for analyses (37 were lost to follow‐up). Study design, statistical analyses and descriptive analysis of the patients' cohort at baseline are detailed in Supporting Information S1.

Intraclass correlation coefficient was 0.594, with a 95% confidence interval of [0.451–0.708] (*p* < 0.001) when comparing ADAS7 values with SCORAD. Using a cut‐off value of ≥18 (as defined and validated in the preliminary study^5^) to detect moderate‐to‐severe patients, positive and negative predictive values were 88.7% and 76.3%, respectively. Sensitivity and specificity were 85.9% and 80.5%, respectively. For a subset of 40 patients for whom long‐term data were available, median ± interquartile range (IQR) ADAS7 scores were calculated and presented on boxplots to illustrate disease activity over a period of 15.5 ± 15.7, [9.2–25.0] weeks (median ± IQR, [quartile [Q]1 − Q3]) (Figure 2). Patients had “active” disease when their IQR value was >8.875 (median of the distribution of all patients' IQR values, used as threshold value—detailed demonstration of variability is provided in Supporting Information S1). Of these, 11/20 (55.0%) changed severity category (i.e., their IQR overlapped two categories) in contrast to 4/20 (20.0%) patients with non‐active disease.

**FIGURE 2:**
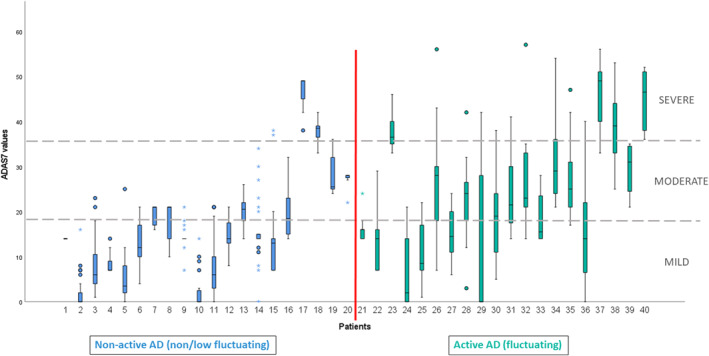
Disease activity illustrated in boxplots for 40 patients over a period of (median ± IQR, Q1–Q3) 15.5 ± 15.7 [9.2–25.0] weeks. Each boxplot graphically represents the distribution of ADAS 7 values, by displaying minimum, maximum, first/third quartiles and median (horizontal line), for each patient. Circles represent mild outliers (values that are more than 1.5 × IQR below Q1 or above Q3) and asterisks the extreme outliers (values that are more than 3.0 × IQR below Q1 or above Q3). Red vertical line divides the cohort into “non‐active” AD group if IQR <8.875 and “active” AD group if IQR >8.875. Mild AD: 0–17; moderate AD 18–36; severe AD 37–56. AD, atopic dermatitis; IQR, interquartile range; Q, quartile.

Effective management of AD patients and treatment adaptations must be based on reliable outcomes that reflect disease activity. With the emergence of new systemic immunomodulatory therapies, long‐term disease control and potential prolonged remission with anti‐IL4Rα treatment represent future therapeutic challenges.^4^ ADAS7 correlated with SCORAD with good sensitivity and specificity, demonstrating that it can be used to reliably determine a patient's disease severity at a given time‐point. Additionally, ADAS7 enabled assessment of AD activity, by identifying (i) controlled AD patients (low median ADAS7 scores with IQR <8.875 and few outlier values); (ii) non‐controlled AD patients with continuously severe disease (high median ADAS7 scores with IQR <8.875 and few outliers); (iii) non‐controlled AD patients with active (high fluctuating) disease (IQR >8.875). This score identified one third of patients who changed severity category during the study, emphasizing the need for clinical scoring that effectively assesses the dynamic aspect of the disease.^6^ The concept of flare patterns and correlation of flare patterns to disease severity is more and more highlighted as patients with more frequent and severe flares tend to have worse quality of life and low treatment satisfaction.^7^ Furthermore, ADAS7 addresses the need of an AD patient‐oriented definition that accounts for not only skin symptoms but also emotional and social implications.^8^

The limitations of this study are: (i) ADAS7 correlation analysis was only made with SCORAD, the only score assessing both clinical signs and patient‐reported symptoms; (ii) the sample size of 40 patients may limit generalization of the findings to the broader population of AD patients; (iii) approximatively a third of the baseline population was lost‐to‐follow‐up, which could lead to selection bias if these patients have different characteristics compared to those who completed the study, and (iv) there may be unmeasured confounding factors influencing the outcomes, such as variations in environmental exposures, comorbidities, or concurrent treatments that were not controlled for in the analysis.

ADAS7 is a patient‐reported score that combines all outcomes recommended by the HOME group. With the assessment of disease activity over several weeks, it represents an innovative tool for discussion with the patient regarding their long‐term disease control and the adaptations of therapeutic strategies.

## AUTHOR CONTRIBUTIONS

**Axel De Greef**: Conceptualization; data curation; formal analysis; funding acquisition; investigation; methodology; writing—original draft; writing—review and editing. **Alexia Degraeuwe**: Conceptualization; data curation; investigation; methodology. **Nina Nielens**: Conceptualization; data curation; investigation; methodology. **Anne‐Sophie Darrigade**: Data curation; investigation. **Céline Bugli**: Conceptualization; methodology. **Laurence de Montjoye**: Conceptualization; formal analysis; investigation; methodology; supervision; writing—review and editing. **Marie Baeck**: Conceptualization; formal analysis; investigation; methodology; supervision; writing—review and editing.

## CONFLICT OF INTEREST STATEMENT

The authors declare no conflicts of interest.

## Supporting information

Supporting Information S1

## Data Availability

Dataset generated during and/or analyzed during the current study are available from the corresponding author on reasonable request.
