# Supplementary material for: Atopic Dermatitis Activity Score 7 (ADAS7): A tool for disease activity assessment
Source: Clin Transl Allergy. 2024 Sep 12;14(9):e12393. doi: 10.1002/clt2.12393 (PMC11392654; doi:10.1002/clt2.12393)
Supplement: Supplementary file 1 — Supporting Information S1 [file CLT2-14-e12393-s001.docx]

**Supporting information**

**1. Descriptive analysis of the patients’ cohort**

| **Variable** | **Value** |
| --- | --- |
| Patients, n total  Median age, years (± IQR, [Q1,Q3])  Sex, female, n/n total (%) SCORAD at baseline, median (± IQR, [Q1,Q3]) ADAS7 seven days after inclusion, median (± IQR, [Q1,Q3])  Atopic comorbidities, n/n total (%)     Allergic asthma     Allergic rhinoconjunctivitis     Food allergy  Previous topical treatments for atopic dermatitis, n/n total (%)     Emollients     Topical corticosteroids     Topical immunomodulators  Not available  Previous systemic treatments for atopic dermatitis, n/n total (%)     Corticosteroids     Cyclosporine     Phototherapy     Methotrexate     Dupilumab   Tralokinumab     Upadacitinib  Others^a^  Not available | 137 28.0 (± 19.2, [21.0, 40.2]) 75/137 (54.7) 39.7 (± 26.3, [26.3, 52.6]) 24 (± 15.5, [18.7, 34.2]  59/137 (43.1) 79/137 (57.7) 30/137 (21.9)  124/135 (91.8) 127/135 (94.1) 60/135 (44.4) 2/137 (1.4)  34/135 (25.2) 27/135 (20.0) 21/135 (15.5) 3/135 (2.2) 4/135 (2.9) 1/135 (0.7) 1/135 (0.7) 13/135 (9.6) 2/137 (1.4) |

*Abbreviations*: ADAS7, Atopic Dermatitis Activity Score 7; IQR, interquartile range; SCORAD, SCORing Atopic Dermatitis; Q, quartile.
^a^Other treatments: azathioprine (n=1), mycophenolate mofetil (n=1), omalizumab (n=1), anti-histamines (n=10)

**2. Patients and Methods**

2.1 Inclusion/exclusion criteria

The inclusion criteria were: diagnosis of atopic dermatitis (AD) by a dermatologist according to the revised Hanifin and Rajka criteria, topical preventive or curative treatment of AD and/or systemic treatment of AD, AD in remission or flare, acute or chronic AD, no SCORing Atopic Dermatitis (SCORAD) cutoff, no age restriction.

The exclusion criterion was: non-atopic eczema.

2.2 Course of the study

The inclusion visit (V1) was followed by follow-up visits at 2 (V2) and 6 (V3) months.

*2.2.1. At baseline, inclusion visit (V1)*

By the investigator:

- Completion of consultation form (including atopic comorbidities), details of previous treatment for AD

- Completion of the SCORAD

*2.2.2. Follow-up consultation at 2 months (V2)*

By the patient:

- Return of the ADAS7 score sheets filled out daily by the patient

By the investigator:

- Filling in the consultation form with details of the ADAS7 score week by week

- ADAS7 score stored in computer file

- Completion of SCORAD

*2.2.2. Follow-up consultation at 6 months (V3)*

By the patient:

- Return of the ADAS7 score sheets filled out daily by the patient

By the investigator:

- Filling in the consultation form with details of the ADAS7 score week by week

- ADAS7 score stored in computer file

- Completion of SCORAD

2.2 Atopic Dermatitis Activity Score 7 (ADAS7) – practical aspects

The score is calculated over 1 week (figure 1). The patient (or the parents if the patient is < 8 years) fills in the daily chart for the two items: (i), extension of the eczema, titled “Lesions” and (ii), itch, pain, or social impairment, titled “Itch/Pain/Discomfort”. Each item is filled with a score from 0 to 4, 0 equivalent to “no symptoms” and 4 to “severe symptoms”. To evaluate the extension of the AD, the patient must determine the dryness, the redness, or crusts with a cutoff of 5 palms of their own hand, to facilitate the evaluation of the body surface area affected. For the second item, the patient must determine the impact of itching on their daily activities, sleep, and social relationship. The chart is presented with a legend characterizing each severity score (from 0 to 4). A weekly score is calculated by addition of the daily scores. The minimal ADAS7 score is 0, and the maximum is 56. Mild disease is <18, moderate disease is between 18 and 36, and severe disease is between 37 and 56. The value of 18 was chosen by dividing the total score into three equal parts of clinically meaningful difference.

2.3 Statistical analyses

Statistical analyses were performed using SPSS software v28.0.1.1 (IBM Corporation, Armonk, NY, USA)

*Demonstration of correlation*

To assess the agreement between the ADAS7 and SCORAD, we computed concordance using intraclass correlation coefficient (ICC) with random factorial design. Test retest reliability between pooled SCORAD scores obtained at V1 and V2 and pooled ADAS7 scores obtained the week after V1 and/or the week before V2 was assessed using an ICC as measure of absolute agreement with a two-way mixed model.

| **Pooled SCORAD scores** | **Pooled ADAS7 scores** |
| --- | --- |
| SCORAD at V1 (60 patients^a^)  and  SCORAD at V2 (40 patients) | ADAS7 the week after V1 (60 patients^a^)  and  ADAS7 the week before V2 (40 patients) |
| Range, 0.0-84.5 | Range, 0.0-50.0 |
| ^a^Data for 52 of them were retrieved from the preliminary study already published^[[1]](#footnote-1)^ | |

*Demonstration of variability*

Prospectively collected ADAS7 weekly values from V1 to V3 were available for a subgroup of 40 patients, who filled the chart during a period of 15.5 ± 15.7 weeks (median ± interquartile range [IQR], quartile [Q]1, Q3 of 9.2, 25.0). Median ADAS7 value ± IQR was calculated for each patient. Patients were then sorted into two equal groups after defining the median of the distribution of all patient IQR values (i.e., 8.875) as the threshold value. (“non-active” AD group if IQR < 8.875 and “active” AD group if IQR > 8.875), and attributed a number between 1 to 40.

1. Darrigade AS, Colmant C, de Montjoye L, et al. Atopic Dermatitis Score 7 (ADS7): A promising tool for daily clinical assessment of atopic dermatitis. *Allergy*. 2020;75(5):1264-1266. doi:10.1111/all.14104 [↑](#footnote-ref-1)
